# Application of Deep Learning in Neuroradiology: Brain Haemorrhage Classification Using Transfer Learning

**DOI:** 10.1155/2019/4629859

**Published:** 2019-06-03

**Authors:** Awwal Muhammad Dawud, Kamil Yurtkan, Huseyin Oztoprak

**Affiliations:** Department of Computer Engineering, Cyprus International University, Nicosia, Cyprus

## Abstract

In this paper, we address the problem of identifying brain haemorrhage which is considered as a tedious task for radiologists, especially in the early stages of the haemorrhage. The problem is solved using a deep learning approach where a convolutional neural network (CNN), the well-known AlexNet neural network, and also a modified novel version of AlexNet with support vector machine (AlexNet-SVM) classifier are trained to classify the brain computer tomography (CT) images into haemorrhage or nonhaemorrhage images. The aim of employing the deep learning model is to address the primary question in medical image analysis and classification: can a sufficient fine-tuning of a pretrained model (transfer learning) eliminate the need of building a CNN from scratch? Moreover, this study also aims to investigate the advantages of using SVM as a classifier instead of a three-layer neural network. We apply the same classification task to three deep networks; one is created from scratch, another is a pretrained model that was fine-tuned to the brain CT haemorrhage classification task, and our modified novel AlexNet model which uses the SVM classifier. The three networks were trained using the same number of brain CT images available. The experiments show that the transfer of knowledge from natural images to medical images classification is possible. In addition, our results proved that the proposed modified pretrained model “AlexNet-SVM” can outperform a convolutional neural network created from scratch and the original AlexNet in identifying the brain haemorrhage.

## 1. Introduction

Intracranial haemorrhage (ICH) reveals as a bleeding within the intracranial vault [1]. Weak blood vessels, hypertension, trauma, and drug abuse are generally what trigger such a medical condition. ICH is a neurologic emergency in which it can have several subtypes such as basal ganglia, caudate nucleus, or pons. The types of haemorrhage are generally dependent on the anatomic location of bleeding [2]. According to the American Heart Association and American Stroke Association, the early and timely diagnosis of ICH is significant as this condition can commonly deteriorate the affected patients within the first few hours after occurrence [3]. Noncontrast head computer tomography (CT) is the imaging modality used to detect haemorrhage due its wide availability and speed. This modality has shown a high sensitivity and specificity in detecting acute haemorrhage [2].

Recently, deep learning has risen rapidly and effectively. Deep learning-based networks have shown a great generalization capability when applied to solve challenging medical problems such as medical image classification [4, 5], medical image analysis [6], medical organs detection [7], and disease detection [8]. Convolutional neural networks were the most effective networks among deep networks, for they own the paradigms of more biologically inspired structures than other traditional networks [9].

Eventually, various convolutional neural networks were developed such as AlexNet [10], VGG-NET [11], and ResNet [12]; these deep networks are all extensively trained on a large database named ImageNet, Large-Scale Visual Recognition Challenge [13], and they were considered as the state of the art in image classification [11–13]. These networks are considered as machine learning methods that can learn features hierarchically from lower level to higher level by building a deep architecture of the input data.

The rise in deep convolutional neural networks performance, due to their abstractions of different levels of features, motivated many researchers to transfer the knowledge acquired by these networks, when trained on millions of images into new tasks such as medical image classification, to benefit from their learned parameters, in particular, weights.

These convolutional neural networks models use fully connected layers, which represent a feedforward neural network trained using the conventional backpropagation algorithm. This means that these models may have the same drawbacks of the conventional simple neural network.

An effective neural network model is the one that performs well during both training and testing datasets; a good balance between variance error and bias error must be struck [14]. For simple models, a high bias and a low variance situation reveals when training these models; that is called underfitting. For more complex neural network models, the progress of training may let the model enter a region of low variance and bias; this can be considered as a good fit. However, as the training progresses further (more complex models), the model may go through a high variance and low bias, that is called overfitting. This is considered a major problem in training a complex neural network model.

There are many approaches for alleviating this problem [15]. These approaches include early stopping, weights penalization, weights pretraining, and dropout of hidden neurons. However, in our study, we ought to avoid these problems by replacing the SoftMax neural network with a multiclass SVM that acts as a classifier for both pretrained employed models. There have been many conducted studies [16–18] that attempt to find an alternative to SoftMax function for classification tasks. All these studies concluded that the support vector machine (SVM) might be the appropriate alternative as it may slightly boost the performance of neural network compared to the conventional SoftMax function.

Thus, in this paper, we aim to transfer the knowledge acquired by AlexNet into a new target task: classifying the CT brain haemorrhage into haemorrhage or nonhaemorrhage images. Moreover, a CNN is created from scratch and a modified AlexNet combined with SVM are also employed to perform the same classification task. The goal of employing one CNN created from scratch and fine-tuning a pretrained model for the same classification task is to show that transfer learning-based network can perform better when data are not much. Also, it is aimed to show that sufficient fine-tuning of a pretrained model can eliminate the need for training a deep CNN from scratch which usually takes long time and requires large number of images to learn. Note that in this research, the CNN created from scratch is denoted as CNN, the pretrained model that uses original AlexNet architecture is denoted as AlexNet, and the modified model is denoted as AlexNet-SVM.

The paper is structured as follows: Section 1 is an introduction of the work.  Section 3 is a brief explanation of the convolutional neural networks basics, while Section 4 explains the transfer learning concept including AlexNet.  Section 5.3 discusses the training of the two employed deep networks in which the data used for training are described.  Section 6 discusses the networks performances and compares the results of both models. Finally, Section 8 is conclusion of the paper.

## 2. Related Work

Convolutional neural networks have been employed to overcome big medical challenges like image segmentation [19] and control for people with disabilities [20]. Hussain et al. [19] have developed a convolutional neural network designed for the segmentation of the most common brain tumor, i.e., glioma tumor. The authors proposed a system composed of two networks, stacked together to form a new ILinear nexus architecture. This new architecture was capable of achieving the best results among all the proposed and related architectures. Another study by Abiyev and Arslan [20] showed that convolutional neural networks can also be used as supporting elements for people with disabilities. The authors proposed a human-machine interface based on two convolutional neural networks designed for disabled people with spinal cord, to control mouse by eye movements. Their work was validated and tested by a handcrafted dataset, and results showed that the network's performance outscored many other related works.

Furthermore, deep learning techniques were employed by Helwan et al. [21] to classify brain computer tomography (CT) images into haemorrhage or healthy. The authors used autoencoders and deep convolutional neural networks to perform this task. As authors claimed, the employed models performed differently when trained and tested on 2527 images. It was found that the stacked autoencoder used in their paper consists of three hidden layers and outperformed other employed networks, where it achieved the highest classification rate and the lowest MSE. The authors concluded that the possible reason of this outperformance on the stacked autoencoder over convolutional neural network is due to the small number of data used for training, as a CNN needs large amount of training examples in order to converge.

In another study by Mahajan and Mahajan [22], brain haemorrhage was examined in more refined manner by feeding using the watershed algorithm along with artificial neural network (ANN) for CT identification of brain haemorrhage type. The authors of this work used features extraction before feeding images to the neural classifier, in which different features were extracted using grey-level co-occurrence matrix (GLCM). Features were then classified by a conventional backpropagation neural network used to identify the type of haemorrhage. They found that adequate image processing techniques such as noise removal and high segmentation methods are required for accurate identification of haemorrhage.

Furthermore, Gong et al. [23] focused on dividing brain CT images into regions, where each region could either be normal or haemorrhage. For images containing haemorrhage, the regions which did not include haemorrhage were treated as normal regions resulting in a highly imbalanced dataset. The researcher had utilized an image segmentation scheme that used ellipse fitting, background removal, and wavelet decomposition technique. The weighted precision and recall value for this approach were approximately 83.6% and 88.5%, respectively.

## 3. Convolutional Neural Network

**Figure 1:**
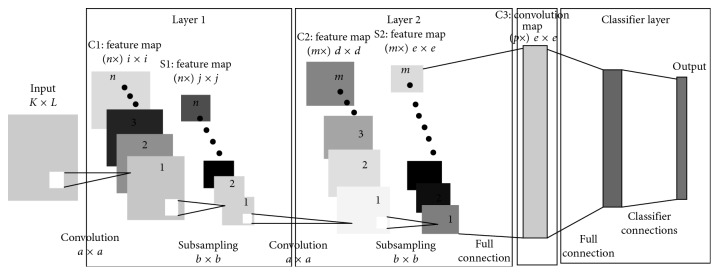
Convolutional neural network.

Convolutional neural network (CNN) is a well-employed network for several tasks in machine vision and medicine [24, 25]. Generally, the CNN relies on architectural features which include the receptive field, weight sharing, and pooling operation to take into account the 2D characteristic of structured data such as images [26]. The concept of weight sharing for convolution maps drastically reduces model parameters; this has the important implications that the model is less prone to overfitting as compared to fully connected models of comparable size. The pooling operation essentially reduces the spatial dimension of input maps and allows the CNN to learn some invariance to moderate distortions in the training; this feature enhances the generalization of the CNN at test time as the model is more tolerant to moderate distortion in the test data [27]. The typical CNN is shown in Figure 1. Essentially, convolution layers, pooling layers, and the fully connected layers are shown. For example, layer 1 employs *n* convolution filters of size *a *×* a* to generate a bank of *n* convolution maps (C1) of size *i *×* i*; this is followed by a pooling (subsampling) operation on the convolution maps with a window size of *b *×* b*. Therefore, the pooling layer (S1) composes *n* feature maps of size *j *×* j*, where, *j* = *i*/*b* [25]. The convolution layer performs feature extraction on the incoming inputs via a convolution filter of specified size. The pooling operation pools features across input maps using a window of specified size; common pooling operations used in applications are the average and max pooling [28]. In average pooling, the average value of the inputs captured by the pooling window is taken, while, in max pooling, the maximum value of the inputs captured by the pooling window is taken. For learning the classifier model, features are forward-propagated through the network to the fully connected layer with an output layer of units. Then, the backpropagation learning algorithm can be employed to update the model parameters via the gradient descent update rule [29].

## 4. Transfer Learning

In medical image analysis and processing, a most common issue is that the number of available data for research purposes is limited and small. Hence, training a fully deep network structure like CNN with small number of data may result in overfitting, which is usually the reason of low performance and generalization power [30]. Transfer learning is a solution to this problem where the learned parameters of effective and well-trained networks on a very large dataset are shared. The concept of transfer learning is the use of a pretrained model that is already trained on large datasets and transfers its pretrained learning parameters, in particular weights, to the targeted network model. To be able to use the network for another problem, the last fully connected layers are then trained with initial random weights on the new dataset. Although the dataset is different than the one that the network was trained on, the low-level features are similar. Thus, the parameters' transfer of the pretrained model may provide the new target model with a powerful feature extraction capability and reduce its training computations and memory cost. Transfer learning has been used extensively in medical imaging, and it showed a great efficacy in terms of accuracy, training time, and error rates [10, 31, 32]. In this paper, we present a modified pretrained model, AlexNet, that has been employed for the classification of CT brain haemorrhage images into normal and abnormal classes.

### 4.1. AlexNet

AlexNet is the first convolutional neural network that achieved the highest classification accuracy at the ImageNet Large Scale Visual Recognition Challenge (ILSVRC) in 2012 [10]. This deep structure is comprised of eight main layers; the first five layers are mainly convolutions, while the last three are fully connected layers. Each convolutional layer is followed by an activation function layer, i.e., rectified linear units layer (ReLU), proposed to improve the performance of the network by making the training faster than equivalents of “tanh” activation functions [10]. After each convolution layer, a max pooling is used in AlexNet, in order to reduce the network size. Moreover, a dropout layer is added after the first two fully connected layer which helps to reduce the number of neurons and prevent overfitting [33]. Finally, a layer is added after the last layer to classify the input given data. Figure 1 shows the structure of the AlexNet.

## 5. Materials and Methods

This work addresses the problem of the classification of the CT brain images into normal or haemorrhage, which can be a hard task for some junior radiologists and doctors. The problem is addressed by the implementation of a deep learning network trained extensively to acquire the power of extracting low to high levels of features from normal brain CT images and others with haemorrhage medical conditions using its designed and trained filters. These features are then what distinguishes the class of the brain images, i.e., haemorrhage or not. Nonetheless, the transfer of knowledge from original to target task, which is here Haemorrhage identification, is also considered by transferring the knowledge of a pretrained model known as AlexNet, into a new classification task and testing it by the same number of images used for testing the CNN created from scratch. In this manner, we aim to address the central issue in medical image analysis and diagnosis: training deep CNN from scratch is not needed; instead, use a pretrained modified AlexNet by adding SVM classifier to transfer its knowledge to a new target task with sufficient fine-tuning. Our conducted experiment on the CT brain haemorrhage classification using a CNN created from scratch and the pretrained models will demonstrate the truth and accuracy behind this central issue.

### 5.1. Data

The two employed models are trained and tested using normal and diseased brain computer tomography (CT) images collected from the Aminu Kano Teaching Hospital, Nigeria [34]. It is important to note that the abnormal images collected from this database are of different types of haemorrhage, but they were all labeled as haemorrhage, because this work aims to classify whether the CT slice contains haemorrhage or not; haemorrhage identification from set of images regardless of the haemorrhage pathology type it may have is feasible [35].

### 5.2. Data Augmentation

Deep networks are data-hungry systems [36], hence the more data you feed them, the more powerful and accurate they become. Therefore, in this work we decided to use data augmentation in order to multiply the number of images collected for the database, which can help in preventing the overfitting that may be encountered during training [37]. Thus, each image is first rotated left and right and then flipped 70, 160, and 270 degrees. Overall, a total number of 12635 normal and haemorrhage CT brain images are obtained. Note that 70% of the data are used for training the employed networks while 30% are used for testing, i.e., 8855 and 3790 images, respectively.  Table 1 shows the learning scheme that is used in this work.

**Figure 2:**
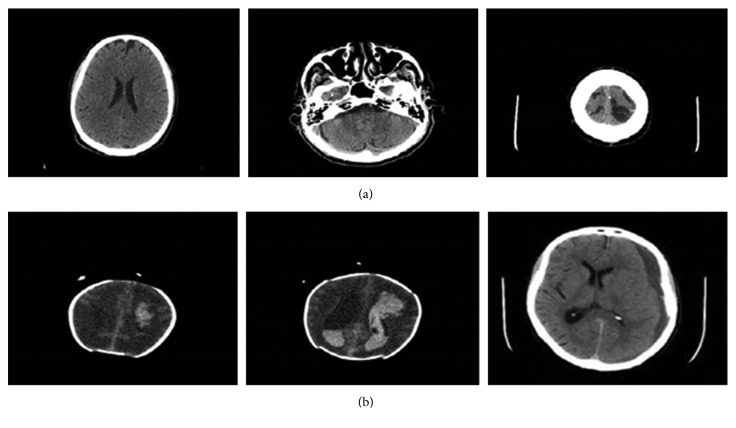
Sample of the databases training and validating images. (a) Haemorrhage images; (b) normal images.

Figure 2 shows some normal and haemorrhage CT slices of the brain that are the used for training and testing the deep networks.

The images of this database are originally of size 1024 ∗ 1024 ∗ 1 pixels; hence, they were first downsampled to 227 ∗ 227 ∗ 1 pixels to fit the input layer of the pretrained model: AlexNet which does not accept other input data sizes. Note that we decided to use the same input images size for the CNN created from scratch, only for networks performance comparison purposes, although any size could be used. Moreover, the images of the database are of grayscale type, and since the AlexNet model requires 3-channels input data, images were all converted to RGB by concatenating their grayscale channel for three times to become 227 ∗ 227 ∗ 3.

### 5.3. Training the Network Models

The two employed deep models are simulated using MATLAB environment. The networks were trained on a Windows 64-bit desktop computer with an Intel Core i7 4770 central processing unit (CPU) and 16 GB random access memory. It is important to mention that there was no graphical processing unit (GPU) available in the used desktop.

The performance evaluation of the networks was carried out using a held-out test set 30% of the data. The calculation of the loss and accuracy was achieved as follows:

(1) $$\begin{array}{c} \text{Loss}=-\left(\frac{1}{n}\right)\overset{n}{\underset{i=1}{\sum }}\log\;P\left(C\right),\\ \text{Accuracy}=\frac{C}{N}, \end{array}$$

where *P*(*C*) is the probability of the correctly classified images, *n* is the number of images, while *N* is the total number of images during the training and/or testing phases.

#### 5.3.1. CNN Training

The model architecture and training settings for the CNN employed to perform the classification of brain haemorrhage are presented in this section. Extensive tests are performed to determine the best learning parameters that optimize the neural network. Note that out of the retrieved 12635 brain CT images, 8855 images are used for training and 3790 images are used for validating the trained network.

**Figure 3:**
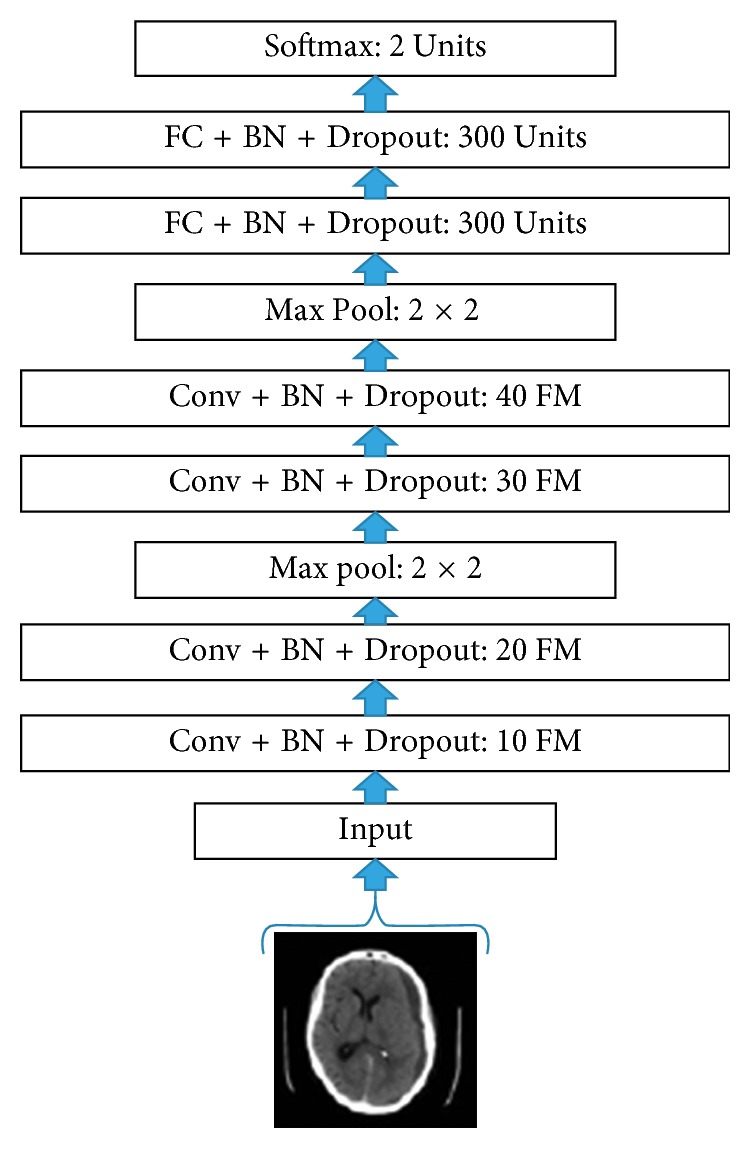
Proposed CNN architecture.

The CNN architecture employed for the classification of brain haemorrhage images is shown in Figure 3, where “Conv” denotes a convolution layer, “BN” denotes batch normalization, “FM” denotes feature maps, and “FC” denotes fully connected layer. In this paper, all convolution operations are performed using convolution filters of size 3 × 3 with zero padding; all pooling operations are performed using max pooling windows of size 2 × 2; the input images to the model are of size 32 × 32.

**Figure 4:**
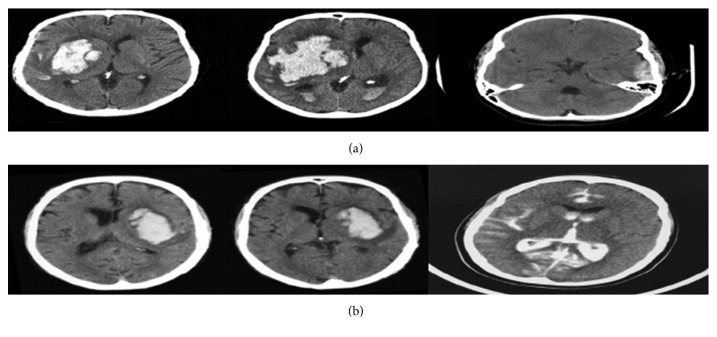
A sample of the brain images collected from the Internet to test the robustness of the system [41].

For designing the proposed architecture, we take into consideration the size of available (i.e., limited) training data for constructing a learning model that is considerably regularized. For example, we employ batch normalization and dropout training schemes which have been shown to improve model generalization [38–40]. For optimizing the proposed model, we employ minibatch optimization via gradient descent; we use a batch size of 60. In addition, we use a learning rate of 0.001 and train the model for 100 epochs. The learning curve for the trained CNN is shown in Figure 4; a validation accuracy of 90.65% is achieved.

**Figure 5:**
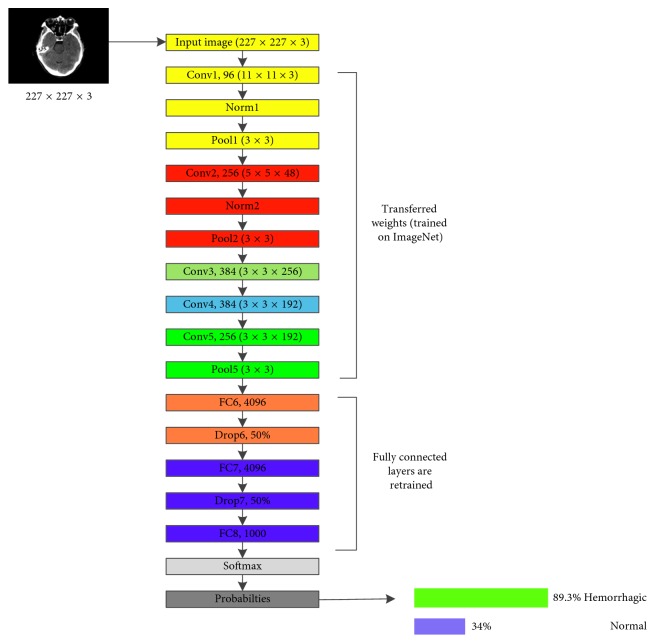
AlexNet proposed transfer learning network for the haemorrhage classification.

In addition, we observe a slight drop in validation performance when dropout and batch normalization are not employed for training the model; a validation accuracy of 87.33% is obtained. The overall proposed system for brain haemorrhage identification is tested using few CT brain haemorrhage images obtained from different sources available online. From the aforementioned database, we collect CT brain images of subjects with different haemorrhage conditions as test images. i.e., Figure 5. Experimental results show that the developed haemorrhage identification deep framework is capable of effectively classifying the haemorrhage within the test images with an accuracy of 87.13%.

We note that in contrast to other works that train and test the proposed approach on the same dataset, the proposed pipeline in this paper has been trained and validated on one dataset and achieved promising results when tested again on a completely different dataset. This shows the robustness of the deep CNN that is designed for such classification task.

#### 5.3.2. AlexNet Training

AlexNet is the pretrained model selected to be used in this research because of its effective power in feature extraction. As can be seen in Figure 5, this deep convolutional neural network is comprised of 5 convolutional layers denoted as CONV1 to CONV5. These layers are followed by 3 fully connected layers denoted as FC1 to FC3, along with a Softmax activation function in the output layer (multinomial logistic regression).

In this research, the publicly available weights of the network trained against the ILSVRC12 are used. As a pretrained model is employed (AlexNet), the final fully connected layer (FC8) was disconnected in order to add a new layer having 2 output neurons corresponding to the two CT brain images' categories. Note that the weights of this layer are initialized at random.

Contrarily, the remaining five convolutional layers are kept in the network for sharing the learned parameters, in particular, weights. These weights are already trained on large datasets, ImageNet, to extract high-level features of the input data. Thus, when transferring the knowledge of AlexNet to haemorrhage classification task, these weights can act as a powerful extractor of different levels of abstractions from input data features.

The network is trained using minibatch of size 200 images of each iteration via stochastic gradient descent SGD [42]. Also, an initial learning rate is set to 0.01 to the fully connected layers (FC6, FC7, and FC8) and a reducing factor of 0.1 after 2000 iterations. Wherefore, this may fasten the learning of the network for the final fully connected layer (FC8).  Table 2 shows the networks parameters during training and the result of the classification task. As seen, AlexNet has reached average training and testing accuracy of 94.12% and 92.13%, respectively.

An image from the test dataset is selected to evaluate the performance of the network in the classification pathway.  Table 3 shows the mean square error (MSE) loss after each convolutional layer being trained.

#### 5.3.3. Proposed AlexNet-SVM Training

**Figure 6:**
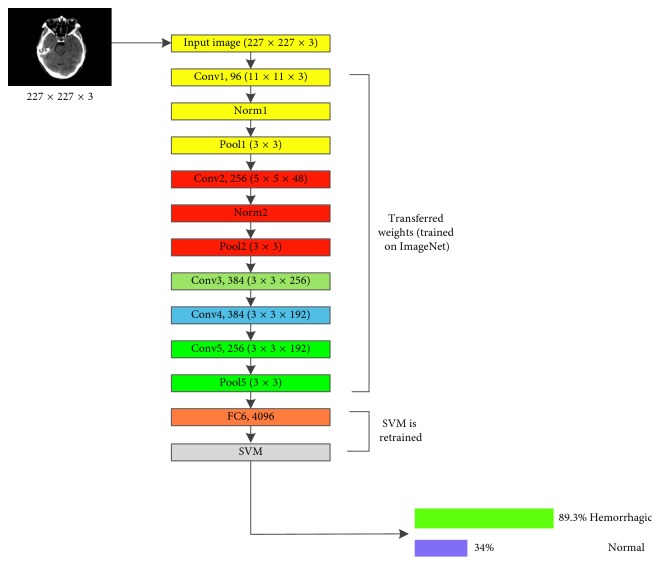
Modified AlexNet (AlexNet-SVM).

Figure 6 shows the architecture of the modified version of AlexNet, in which an SVM classifier is used instead of a neural network. Similarly, this modified network, AlexNet-SVM, is also trained with the same conditions and same number of images except for the number of iterations which is here 140.

As seen in Figure 6 AlexNet-SVM's training parameters were similar to the parameters of AlexNet; however, it is noted that their performance was different. AlexNet-SVM was trained and it reached a lower MSE (0.054) compared to other networks. In addition, AlexNet-SVM achieved higher accuracies during training and testing with values of 96.34% and 93.48%, respectively.

## 6. Results and Discussion

**Figure 7:**
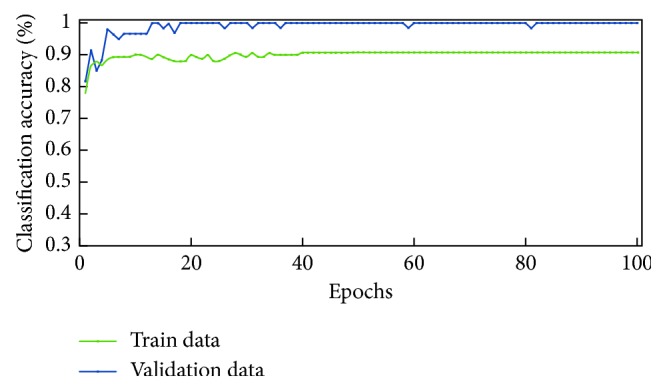
Learning curve for the trained CNN.

**Figure 8:**
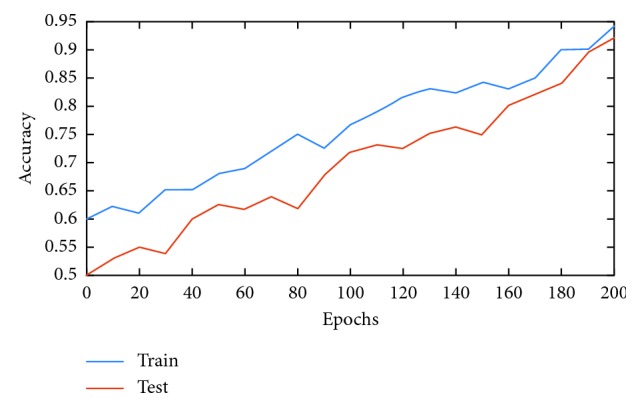
Learning curves of AlexNet.

**Figure 9:**
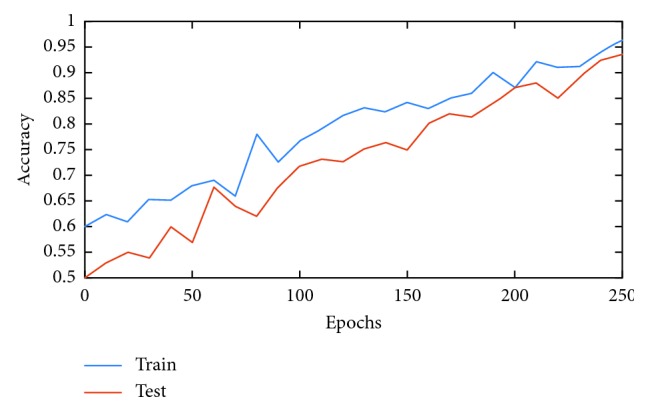
Learning curves of AlexNet-SVM.

**Figure 10:**
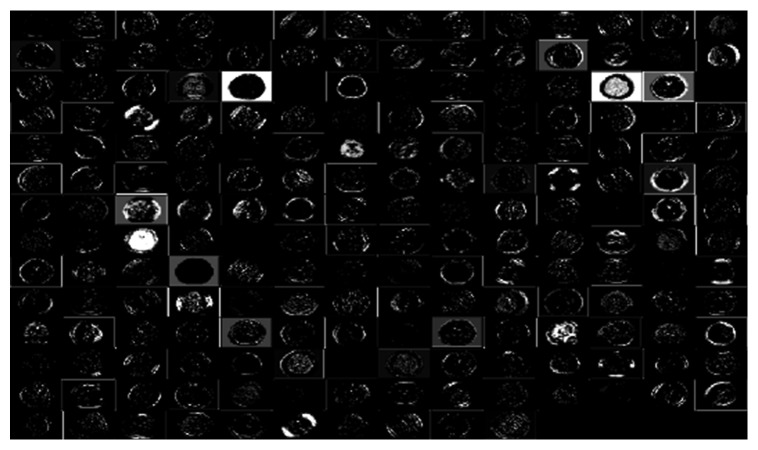
Learned kernels of CNN.

**Figure 11:**
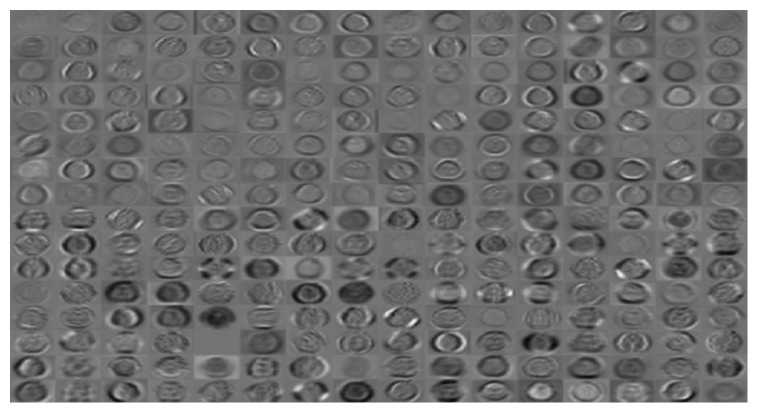
Learned kernels of AlexNet.

Once trained, all network models are tested on 30% of the available data.  Table 4 shows the performances of each model during testing. As can be seen, the CNN, AlexNet, and AlexNet-SVM achieved different accuracies of 90.65%, 92.13%, and 93.48%, respectively. AlexNet-SVM was capable of achieving more accurate generalizing power on unseen data. However, a larger number of epochs was required to achieve such accuracy, which is relatively higher than that needed for CNN and AlexNet to achieve their highest accuracy. It is also noted that AlexNet-SVM reached a lower mean square error (MSE) (0.054) than that reached by AlexNet (0.087) and CNN (0.092); however, this also required longer training time. The learning curves of the trained models are shown in Figures 7–9. The figures show the variations accuracy with respect to the increase of the number of epochs. Consequently, it is seen that all models are trained well, but the increase of depth of AlexNet and AlexNet-SVM makes it more difficult to train, i.e., it required longer time and more epochs to reach the minimum square error (MSE) and converge. Furthermore, it is important to mention that due to this difference in time and epoch number, the classifier of AlexNet-SVM resulted in a lower MSE and higher recognition rate than that scored by AlexNet and CNN. As a result, to understand the learning performance of networks, we have an insight into the different levels features learned by the employed models, by visualizing the learned kernels or features in the convolutional layers, shown in Figures 10 and 11.

Figures 10 and 11 show the learned features of CNN and AlexNet, respectively. From Figure 6, it can be seen that neurons in the first convolution layer are the mostly active neurons in capturing good features in the training data. However, from Figure 11, it is seen that the neurons of the last convolutional layer of AlexNet are the most active neurons in capturing descriptive and different levels features. In addition, compared to CNN, this layer has an improved activity as observed in the learned features. Lastly, it can be noted that the neurons of the first and last convolutional layers of both networks have learned different and interesting representation of the input images. Generally, networks that tend to learn more descriptive and different levels features tend to perform better at run time, as the good knowledge acquired in the unsupervised pretraining contributes to better fine-tuning and classification.

Table 5 shows a comparison of the developed networks with some previous works that were proposed to classify brain haemorrhage using deep learning. Note that we ought to compare our approach with the deep networks and pretrained model researches that provide explicitly achieved accuracies and number of data. Firstly, a general analysis of the table shows that the pretrained models (transfer learning-based networks) achieved higher accuracies when compared to those that were created from scratch. The proposed AlexNet_SVM employed in this research achieved more powerful generalization capabilities than other AlexNet that use neural network classifiers like the networks employed in this research and also in other researches [43]. Moreover, AlexNet-SVM outperformed the networks that were created from scratch such as convolutional neural networks and autoencoders [21]. Furthermore, it is seen that the employed pretrained model (AlexNet) achieved a higher recognition rate (92.13%) than other earlier research works such as CNN created from scratch on less number of images [21]. Also, this model has outperformed other types of deep networks such as autoencoder (88.3%) and stacked autoencoder (90.9%) [21]. This can probably be due to the deficiency of newly born networks in extracting the important features from input images which is a result of the small number of images used for training them in addition to their depth.

Overall, the application of pretrained models to solve haemorrhage classification challenge can end up with satisfying results since these deep structures have gained powerful feature extraction capabilities as they were trained using huge databases such as ImageNet [13]. The obtained results of applying the proposed AlexNet-SVM, AlexNet and CNN in this research show that applying deep CNNs to the problem of brain haemorrhage is promising, in a way that a haemorrhage can be identified by a deep neural network with low margins of error.

### 6.1. Performance Evaluation Metrics

These metrics are derived from classification of the tested sampling images, as shown in Table 6, being derived by a contingency table which is called confusion matrix [13]. Accuracy indicates the percentage of rightly classified image samples, without considering their class labels. For a binary classification that concludes on positive and negative classes, sensitivity is the percentage of correctly classified samples and specificity is the number of correctly negative samples classified:

(2) $$\begin{array}{c} \text{Accuracy}=\frac{\left(\text{TP}+\text{TN}\right)}{\text{TN}+\text{TP}+\text{FP}+\text{FN}},\\ \text{Sensitivity}=\frac{\text{TP}}{\text{TP}+\text{FN}},\\ \text{Specificity}=\frac{\text{TN}}{\text{TN}+\text{FP}}. \end{array}$$

### 6.2. Models Comparison

In this section, the comparison of the conventional AlexNet and the proposed AlexNet-SVM is explained, in order to show the advantages of the fusion of AlexNet and SVM, in addition to the possible reasons of AlexNet-SVM outperformance. As seen in Table 5, the fusion of AlexNet and SVM resulted in a slight boost of accuracy by 0.934. This outperformance is mainly due to the use of a different optimization criterion that the SVM uses. This algorithm is used to minimize the prediction loss on the training set of the neural network. However, in practice, there are two challenges with this risk. First is the convexity; it is not convex which means that many local minimums may exist. Second problem is the smoothness; it is not smooth, which means it may not be practically minimized. In contrast, SVM aims to minimize the generalization error by using structural risk minimization principles for the testing set. As a result of a maximized margin, the generalization ability of SVM is greater than that of the other classifiers.

## 7. Limitations

The effectiveness of deep learning in medical applications is great and improving with time; however, it still encounters some drawbacks, in particular, the availability data. The variability of data (e.g., contrast, noise, and resolution) can be one of the main barriers of the adaptation of deep learning in medicine. These intelligent models can suffer from poor generalization if data contain some noise and when they are generated from different modalities. Moreover, deep learning models are data-driving systems; the more the data, the more efficient they become. The problem is very few data are not publicly available in the medical field due to privacy issues as in most cases, the data contain sensitive information. Thus, we and many other researchers prefer to use transfer learning based models which usually require less number of data to learn, as they are already trained using large amounts of data. Hence, the system is capable of learning different levels of features, which helps in adapting the new task accurately, even if the data are not large.

## 8. Conclusion

In this research, the detection of brain haemorrhage in CT images problem is solved using neural networks and the results sound robust and promising. One of the motivations behind this research is to address and attempt to overcome the difficulties that radiologists might encounter when diagnosing brain haemorrhage suspected images. Hence, we investigated the use of a potential deep convolutional neural network that can help the medical experts in making more accurate decisions. As a result, this may reduce the diagnosis error and boost the accuracy of haemorrhage identification made by medical experts. The paper proposes a pretrained modified network “AlexNet-SVM” for the same classification task. The three models including the proposed model were trained on a relatively small database in order to examine the network performance. It is obvious that the application of deep learning networks in medical image analysis encounters several challenges. The most common challenge is the lack of large training data sets which can be considered as an obstacle. The experiments conducted in this study demonstrated that the transfer of knowledge into medical images can be possible, even though the deep networks are originally trained on natural images. The proposed model using the SVM classifier helps in improving the performance of AlexNet. Moreover, it was manifested that small number of data can be enough for fine-tuning a pretrained model, in contrast to a CNN created from scratch which needs a large number of data to be trained. Thus, the proposed model's performance is an indicator of how transfer learning-based networks can be considered in brain haemorrhage identification.

## Figures and Tables

**Table 1:** Learning scheme of the networks.

Total number of images	
Train	8855
Test	3790
Total	12635

**Table 2:** Models learning parameters.

	CNN	AlexNet	AlexNet-SVM
Learning parameters	Values	Values	Values
Training ratio (%)	80	80	80
Initial learning rates	0.001	0.01	0.01
Number of epochs	100	200	140
Training accuracy (%)	92.89	94.12	96.34
Testing accuracy (%)	90.65	92.13	93.48
Achieved mean square error (MSE)	0.092	0.087	0.054

**Table 3:** Loss at each convolutional layer of CNN.

Layer	CONV1	CONV2	CONV3	CONV4	CONV5
Loss	0.186	0.341	0.412	0.46	0.51

**Table 4:** Performance comparison of the employed networks.

	CNN	AlexNet	AlexNet-SVM
Testing images	3790	3790	3790
Number of correctly classified images	3436	3492	3543
Accuracy (%)	90.65	92.13	93.48

**Table 5:** Performance metrics of the networks.

Network model	CNN	AlexNet	AlexNet-SVM
Accuracy (%)	89	91	93
Sensitivity (%)	90	93	95
Specificity (%)	86	88	90
Misclassified (%)	11	9	7

**Table 6:** Results comparison with earlier works.

Network models	CNN	AlexNet	AlexNet-SVM	AlexNet [43]	CNN [19]	AE [19]	SAE [19]
Number of images	12635	12635	12635	11,088	2527	2527	2527
Accuracy (%)	90.65	92.13	93.48	92	89.6	88.3	90.9

## Data Availability

The brain haemorrhage data used to support the findings of this study may be released upon application to the Aminu Kano Teaching Hospital, Kano, Nigeria, at http://akth.org.ng/index.php/contact.
