# Corrigendum to “Application of Deep Learning in Neuroradiology: Brain Haemorrhage Classification Using Transfer Learning”

**DOI:** 10.1155/2020/4705838

**Published:** 2020-08-28

**Authors:** Awwal Muhammad Dawud, Kamil Yurtkan, Huseyin Oztoprak

**Affiliations:** Cyprus International University, Faculty of Engineering, Department of Computer Engineering, Nicosia, Northern Cyprus, Mersin 10, Turkey

In the article titled “Application of Deep Learning in Neuroradiology: Brain Haemorrhage Classification Using Transfer Learning” [1], the faculty name was missing from the affiliation. The corrected authors' list and affiliation are shown above.
